# Contrasted evolutionary constraints on secreted and non-secreted proteomes of selected Actinobacteria

**DOI:** 10.1186/1471-2164-14-474

**Published:** 2013-07-13

**Authors:** Subarna Thakur, Philippe Normand, Vincent Daubin, Louis S Tisa, Arnab Sen

**Affiliations:** 1 NBU Bioinformatics Facility, Department of Botany, University of North Bengal, Siliguri 734013, India; 2 Ecologie Microbienne UMR5557, Université Lyon 1, Université de Lyon, CNRS, Cedex, Villeurbanne 69622, France; 3 CNRS, Biométrie & Biologie Evolutive UMR5558, Université Lyon 1, Université de Lyon, Cedex, Villeurbanne 69622, France; 4 Department of Molecular, Cellular, & Biomedical Sciences, University of New Hampshire, Durham, NH, USA

**Keywords:** Actinobacteria, Pathogenesis, Purifying selection, Secretome, Symbiosis

## Abstract

**Background:**

Actinobacteria have adapted to contrasted ecological niches such as the soil, and among others to plants or animals as pathogens or symbionts. *Mycobacterium* genus contains mostly pathogens that cause a variety of mammalian diseases, among which the well-known leprosy and tuberculosis, it also has saprophytic relatives. *Streptomyces* genus is mostly a soil microbe known for its secondary metabolites, it contains also plant pathogens, animal pathogens and symbionts. *Frankia,* a nitrogen-fixing actinobacterium establishes a root symbiosis with dicotyledonous pionneer plants. Pathogens and symbionts live inside eukaryotic cells and tissues and interact with their cellular environment through secreted proteins and effectors transported through transmembrane systems; nevertheless they also need to avoid triggering host defense reactions. A comparative genome analysis of the secretomes of symbionts and pathogens allows a thorough investigation of selective pressures shaping their evolution. In the present study, the rates of silent mutations to non-silent mutations in secretory proteins were assessed in different strains of *Frankia*, *Streptomyces* and *Mycobacterium,* of which several genomes have recently become publicly available.

**Results:**

It was found that secreted proteins as a whole have a stronger purifying evolutionary rate (non-synonymous to synonymous substitutions or Ka/Ks ratio) than the non-secretory proteins in most of the studied genomes. This difference becomes statistically significant in cases involving obligate symbionts and pathogens. Amongst the *Frankia*, secretomes of symbiotic strains were found to have undergone evolutionary trends different from those of the mainly saprophytic strains. Even within the secretory proteins, the signal peptide part has a higher Ka/Ks ratio than the mature part. Two contrasting trends were noticed amongst the *Frankia* genomes regarding the relation between selection strength (i.e. Ka/Ks ratio) and the codon adaptation index (CAI), a predictor of the expression rate, in all the genes belonging to the core genome as well as the core secretory protein genes. The genomes of pathogenic *Mycobacterium* and *Streptomyces* also had reduced secretomes relative to saprophytes, as well as in general significant pairwise Ka/Ks ratios in their secretomes.

**Conclusion:**

In marginally free-living facultative symbionts or pathogenic organisms under consideration, secretory protein genes as a whole evolve at a faster rate than the rest and this process may be an adaptive life-strategy to counter the host selection pressure. The higher evolutionary rate of signal peptide part compared to mature protein provides an indication that signal peptide parts may be under relaxed purifying selection, indicative of the signal peptides not being secreted into host cells. Codon usage analysis suggests that in actinobacterial strains under host selection pressure such as symbiotic *Frankia*, ACN, FD and the pathogenic *Mycobacterium,* codon usage bias was negatively correlated to the selective pressure exerted on the secretory protein genes.

## Background

*Frankia* is a taxon comprising nitrogen-fixing actinobacteria that establish a root symbiosis with dicotyledonous plants belonging to 8 plant families [1]. The phylogeny of these bacteria as determined by classic 16S rRNA sequence analysis clusters them into 4 clusters [2]. The genus *Frankia* appears to have emerged from a group of soil and rhizosphere actinobacterial genera [3], many of which are extremophiles such as the thermophilic *Acidothermus*[2], the gamma-radiation resistant *Geodermatophilus*[4] or the compost-inhabiting Antarctic-dwelling *Sporichthya*[5]. *Frankia* exhibits only a few distinct morpho-physiological features including a distinctive wall sugar [6] and unique specialized structures (termed vesicles) that are surrounded by an envelope containing oxygen-impermeable bacteriohopanetetrol, which serves to protect nitrogenase [7].

Little is known about the nature of the symbiotic determinants involved in the actinorhizal symbiosis. On the host plant-side, the SymRK gene, a transmembrane kinase, has been identified and shown to control development of both actinorhizal nodulation and the mycorhizal infection processes [8]. Furthermore, the actinorhizal host plants, *Alnus* and *Casuarina,* have homologs of this whole symbiotic cascade [9]. On the bacterial-side of the symbiosis, the absence of a well-established reliable genetic system has hindered attempts to identify essential genes involved in the process. However, sequence analysis of three *Frankia* genomes representing contrasting host specificity ranges failed to reveal the presence of genes homologous to the *Rhizobium nod* genes [10] or symbiotic islands [11]. These genomes were found to have undergone contrasted evolutionary pressures resulting in marked differences in their size, transposase content and loss-gain of several determinants. Relative to their saprophytic neighbors, *Frankia* genomes have a reduced number of secreted proteins [12] although these predictions have not been consistently confirmed experimentally [13]. This is evocative of a genome-wide strategy to keep a chemical “low-profile” inside host plant cells.

*Streptomyces* genus is emblematic of soil microbes with its rich array of secondary metabolites that have been exploited for a long time with many powerful drugs ever since streptomycin was characterized [14]. Most *Streptomyces* species are described as saprophytes except for a few such as the potato pathogens *S. scabiei* and related species [15] and the human pathogen *S. somaliensis*[16]. There have also been a number of strains recently described as symbionts or commensals but the exact nature of their interaction with their host is still not clear.

*Mycobacterium* genus is better known for its two terrible disease-causing species, *M. tuberculosis*, agent of tuberculosis [17] and *M. leprae*, agent of leprosy [18] as well as a few less known ones such as *M. ulcerans*. Beside these pathogens, there is a number of saprophytic species such as the pyrene-degrading soil *M. vanbaalenii*[19], or the commensal/environmental *M. smegmatis*[20].

Intracellular bacteria interact in an intimate fashion with host cells, thus facing a paradoxical challenge. Their interactions with their cellular environment through secreted proteins and effectors transported through transmembrane systems may trigger a host defense response that they would then need to fight off. Host cells have elaborate sensing systems to detect motifs that are specific for different classes of pathogens and subsequently trigger defense reactions including synthesis of cysteine-rich defensins, oxygen radicals, or toxic aromatics, which would be detrimental to symbionts [21]. Pathogenic microbes have thus evolved in close interaction with their hosts, in a gene-for-gene pattern that effectively restricts the pathogen to a subset of hosts and modulates genetic diversity as a function of host resistance [22]. For certain lineages, *Frankia* has been shown, to have coevolved with its host plants [23], dramatically altering its transcriptome upon symbiosis onset [24], and is thus expected to have underwent pressures at the level of gene composition. One way of monitoring evolutionary pressures on genomes is to follow rates of silent and non-silent mutations [25]. For pathogens, both diversifying (positive) selection [26,27] and purifying (negative) selection [28,29] have been reported. The situation in symbionts has not been extensively studied, except for few brief reports on *Wolbachia* or *Rhizobium*[30,31]. We undertook this investigation on genomes of three important but diverse genera of Actinobacteria to analyze the selection pressures working on them and have also looked into the evolutionary rate of secreted proteins to assess their biochemical adaptations to the environment. The genera include *Frankia*, a predominantly plant symbiont, *Streptomyces*, a group of soil-dwelling mostly free-living actinobacteria with a few pathogens*,* and *Mycobacterium*, which contains both free-living and pathogens.

## Results and discussion

### Background of the strains chosen for the analysis

*Candidatus* Frankia datiscae (FD) is a non-isolated symbiont that forms effective nodules in Rosaceae, Coriariaceae, Datiscaceae [2]. Hundreds of attempts at isolation of the bacteria in pure culture have failed [32] and these strains are thus considered by many as obligate symbionts [33]. The extent of cospeciation is unknown because cross-inoculation assays have yielded conflicting results. *Frankia* CcI3 (CcI) can be isolated and grown in defined media [34], however it belongs to a homogenous clade that in general is difficult to isolate and culture [2]. Historically, several attempts to isolate the *Casuarina* microsymbionts failed or else yielded atypical strains later found to belong to cluster 3 that could not fulfill Koch’s postulates [35]. On the other hand, *Frankia alni* ACN14a (ACN) can be isolated in pure culture and can nodulate *Alnus* and Myricaceae [36]. It is abundant in soils devoid of host plants and will grow well in the rhizosphere of *Betula*, a close relative of *Alnus*[37]. The two *Elaeagnus* isolates, EAN1pec (EAN) and EuI1c (EuI) grow well and rapidly in pure culture, are abundant in soils without host plants, grow rapidly in pure culture and have the most extensive host range that includes Elaeagnaceae, Myricaceae, Casuarinaceae (*Gymnostoma*), Rhamnaceae as well as Datiscaceae and Coriariaceae where they are present as co-inoculants. The range of substrates on which these strains grow is more extensive than that of other groups [1]. Based on the above information and the genome size, we have divided *Frankia* strains into 3 major groups as Group A: Predominantly free-living Facultative symbiont; Group B: Partly free-living Facultative symbiont and Group C: Marginally free-living or obligate symbiont (Table 1).

**Table 1:** **Grouping of *Frankia *strains based on characteristic features**

**Characteristic features**	**EAN1pec**	**Eul1c**	**ACN14a**	**CcI3**	**DG (FD)**
Growth in pure culture	√	√	√	√	≠
Found in soil away from host plant	√	√	√	≠	≠
Lack of cospeciation	√	√	√	≠	-
All closely related lineages can be grown in pure culture	√	√	≠	≠	≠
Genome size (bp)	8815781	8982042	7497934	5433628	5204281
**Grouping**	**A**	**A**	**B**	**C**	**C**

*Mycobacterium* species include both pathogenic as well as non-pathogenic ones. Pathogenic species include *M. leprae* TN, *M. tuberculosis* CDC1551 and *M. ulcerans* Agy99. Non-pathogenic strains include *M. smegmatis* MC2 155 and *M. vanbaaleni* PYR-1*,* both of them fast growing *Mycobacterium* that exist as saprophytes in the environment (Table 2).

**Table 2:** **Grouping of *Mycobacterium *species based on characteristic features**

**Characteristic features**	***vanbaalenii***	***smegmatis***	***ulcerans***	***tuberculosis***	***leprae***
Growth in pure culture	√	√	√	√	≠
Evidence for cospeciation	-	-	-	√	-
Survival outside of the human body	√	√	√	≠	-
Known animal or environmental reservoir	√	√	√^1^	≠	≠^2^
Genome size (bp)	6491865	6988209	5631606	4403837	3268203
**Grouping**	**A**	**A**	**B**	**B**	**B**

1-in wild koalas, possums, alpacas; 2-except for one species: armadillos.

*Streptomyces* species considered in the analysis are *S. coelicolor* A3 (2)*, S. avermitilis* MA-4680, *S. griseus* NBRC 13350, *S. scabiei* 87.22 and *S. somaliensis* DSM. The first three are soil-dwelling saprophytes which are grown in chemostat cultures for the industrial production of various secondary metabolites including a wide range of antibiotics. The last two are either pathogens of a plant (*S. scabiei* 87.22) or of animals (*S. somaliensis* DSM 40738) (Table 3). In all the three Tables (1, 2 and 3), (√) denotes present; (≠) denotes absent and (-) denotes not known.

**Table 3:** **Grouping of *Streptomyces *species based on characteristic features**

**Characteristic features**	***coelicolor***	***avermitilis***	***griseus***	***somaliensis***	***scabiei***
Growth in pure culture	√	√	√	√	√
Pathogen on plants	-	-	-	-	√
Pathogen on animals	-	-	-	√	-
Symbiont with insect	-	-	√	-	-
Genome size (bp)	9054847	9119895	8727768	5176903	10148695
**Grouping**	**A**	**A**	**A**	**B**	**B**

### Core genome

For the five *Frankia* genomes examined, a core *Frankia* genome of 982 genes was identified. Since the *Frankia* EuI genome was devoid of any *nif* genes, the core genome did not include them. This *Frankia* strain will induce nodule formation on its host plant, *Elaeagnus umbellata,* but produces ineffective nodules that are unable to fix nitrogen [38]. Amongst the *Mycobacterium* genomes, 665 genes were identified as belonging to their core genomes. Since the *Mycobacterium leprae* genome is undergoing reductive evolution, its inclusion in the analysis may have resulted in a considerable decrease in the number of genes in the core genome for the *Mycobacterium*. The five genomes of *Streptomyces* contain 1304 genes in the core genome. Table 4 shows the Average Ka/Ks values of all of the gene orthologs belonging to the core genome.

**Table 4:** Average Ka/Ks value of all the orthologous genes belonging to the core genome

**Strain**	***vs. *Strain**		**Ka/Ks**	**Ka**	**Ks**
*Frankia* ACN					
	*Frankia* CcI		0.047	0.092	6.458
	*Frankia* EAN		0.037	0.15	24.059
	*Frankia* FD		0.034	0.187	31.731
	*Frankia* Eul		0.034	0.18	33.03
		Average	0.038	0.152	23.82
*Frankia* CcI					
	*Frankia* ACN		0.047	0.092	6.458
	*Frankia* EAN		0.033	0.153	24.232
	*Frankia* FD		0.034	0.191	28
	*Frankia* Eul		0.029	0.186	33.82
		Average	0.036	0.156	23.128
*Frankia* FD					
	*Frankia* CcI		0.034	0.191	28
	*Frankia* ACN		0.034	0.187	31.731
	*Frankia* Eul		0.028	0.205	39.126
	*Frankia* EAN		0.029	0.191	36.336
		Average	0.031	0.194	33.798
*Frankia* EAN					
	*Frankia* CcI		0.033	0.153	24.232
	*Frankia* ACN		0.037	0.15	24.059
	*Frankia* Eul		0.031	0.184	39.412
	*Frankia* FD		0.029	0.191	36.336
		Average	0.033	0.169	31.01
*Frankia* Eul					
	*Frankia* CcI		0.029	0.186	33.82
	*Frankia* ACN		0.034	0.18	33.03
	*Frankia* EAN		0.031	0.184	39.412
	*Frankia* FD		0.028	0.205	39.126
		Average	0.031	0.189	36.347
*M. tuberculosis*					
	*M. leprae*		0.089	0.123	2.155
	*M. ulcerans*		0.066	0.097	2.582
	*M. smegmatis*		0.03	0.165	28.61
	*M. vanbaalenii*		0.032	0.166	26.201
		Average	0.054	0.138	14.887
*M. leprae*					
	*M. tuberculosis*		0.089	0.123	2.155
	*M. ulcerans*		0.071	0.134	2.962
	*M. smegmatis*		0.026	0.191	28.417
	*M. vanbaalenii*		0.03	0.196	25.624
		Average	0.054	0.161	14.789
*M. ulcerans*					
	*M. leprae*		0.071	0.134	2.962
	*M. tuberculosis*		0.066	0.097	2.582
	*M. smegmatis*		0.03	0.167	28.253
	*M. vanbaalenii*		0.031	0.169	27.778
		Average	0.05	0.142	15.394
*M. smegmatis*					
	*M. tuberculosis*		0.03	0.165	28.61
	*M. leprae*		0.026	0.191	28.417
	*M. ulcerans*		0.03	0.167	28.253
	*M. vanbaalenii*		0.042	0.128	16.365
		Average	0.032	0.163	25.411
*M. vanbaalenii*					
	*M. tuberculosis*		0.032	0.166	26.201
	*M. leprae*		0.03	0.196	25.624
	*M. ulcerans*		0.031	0.169	27.778
	*M. smegmatis*		0.042	0.128	16.365
		Average	0.034	0.165	23.992
*S. coelicolor*					
	*S. avermitilis*		0.054	0.101	8.594
	*S. griseus*		0.044	0.152	24.613
	*S. somaliensis*		0.041	0.156	27.083
	*S. scabiei*		0.051	0.106	10.949
		Average	0.047	0.135	19.053
*S. avermitilis*					
	*S. coelicolor*		0.054	0.101	8.594
	*S. somaliensis*		0.036	0.152	28.933
	*S. scabiei*		0.057	0.098	7.197
	*S. griseus*		0.041	0.147	25
		Average	0.046	0.132	18.875
*S. griseus*					
	*S. coelicolor*		0.044	0.152	24.613
	*S. avermitilis*		0.041	0.147	25
	*S. somaliensis*		0.041	0.143	21.745
	*S. scabiei*		0.041	0.152	24.843
		Average	0.042	0.149	24.050
*S. somaliensis*					
	*S. coelicolor*		0.041	0.156	27.083
	*S. avermitilis*		0.036	0.152	28.933
	*S. griseus*		0.041	0.143	21.745
	*S. scabiei*		0.035	0.155	28.346
		Average	0.039	0.153	25.597
*S. scabiei*					
	*S. coelicolor*		0.051	0.106	10.949
	*S. avermitilis*		0.057	0.098	7.197
	*S. griseus*		0.041	0.152	24.843
	*S. somaliensis*		0.035	0.155	28.346
		Average	0.045	0.136	19.266

The silent mutation rate (Ks) of all *Frankia* strains was found to range from 6.458 substitution/site between ACN and CcI to 39.412 between EuI and Ean, evocative of saturation. The non-silent rate (Ka) was much lower, ranging between 0.092 substitution/site between ACN and CcI to 0.205 or twice as much between FD and EuI. The Ka/Ks fluctuated in a narrow range of 0.029-0.047, a very low value indicative of a strongly purifying selection, lower than that seen in the *pol* gene of the bovine immunodeficiency virus [39]. This greater than 20-fold difference in mutation rates also illustrates why protein-based phylogenies are better for reconstructing distant relationships than DNA-based ones.

The trends are also more or less similar in *Mycobacterium*. The silent mutation rate in *Mycobacterium* ranges from 2.155 between *M. tuberculosis* and *M. leprae* to 28.61 between *M. tuberculosis* and *M. smegmatis.* The non-silent rate ranges between 0.097 between *M. tuberculosis* and *M. ulcerans* and 0.196 between *M. vanbaalenii* and *M. leprae.* The silent mutation rate of *Mycobacterium* is thus in general much higher than that of *Frankia* while the non-silent rates are comparable between the two taxa. The Ka/Ks fluctuated in a range of 0.026 to 0.089, larger than in *Frankia*.

The silent mutation rate in *Streptomyces* ranges from 7.197 between *S. scabiei* and *S. avermitilis* to 28.933 between *S. somaliensis* and *S. avermitilis.* The non-silent rate ranges from 0.098 between *S. scabiei* and *S. avermitilis* and 0.156 between *S. somaliensis* and *S. coelicolor.* The Ka/Ks fluctuated in a range of 0.035 to 0.057, smaller than in *Mycobacterium* and comparable to that in *Frankia*.

**Figure 1:**
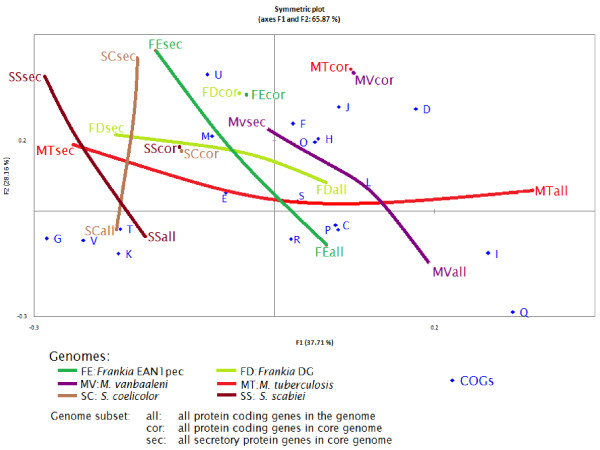
**Factorial correspondence analysis of protein coding genes (all), core genome (cor) and secretory proteins genes in the core genome (sec) for *Frankia *EAN1pec (FE), *Frankia *DG (FD), *S. coelicolor *(SC), *S. scabiei *(SS), *M. tuberculosis *(MT), and *M. vanbaaleni *(MV), within functional COG groups.** The horizontal axis explains 37.7% of the total inertia and the second one 28.2%.

The core secretome (Additional file 1: Table S1) of *Frankia* is represented by 69–89 genes with the nitrogen-fixing symbiotic strains having between 69 and 79 while the non-efficient cluster 4 EuI has 89 genes. The COG categories (besides the poorly defined “R” and “S”) that were mostly represented in the *Frankia* core secretome were “M” (Cell wall/membrane/envelope biogenesis), E (Amino acid transport and metabolism), O (Posttranslational modification, protein turnover, chaperones) and U (Intracellular trafficking, secretion, and vesicular transport). The categories that varied the most between the symbiotic strains and the more saprophytic ones were M and V (Defense mechanisms). *Mycobacterium* had a smaller core secretome of 31–40 genes with the pathogenic *M. leprae* and *M. tuberculosis* having the smallest number of genes. The COG categories that were most abundant were M, E and C (Energy production and conversion). *Streptomyces* had the largest core secretome of the three genera with 72–89 genes. The COG categories that were most represented were M, E, P (Inorganic ion transport and metabolism) and T (Signal transduction mechanisms). A correspondence analysis shows those strains that interact closely with eukaryotic hosts have their secretome positioned close to one another (FD and MT) and away from the more saprophytic strains (Figure 1). Curves joining the genomes as a whole to the secretomes were horizontal in the case of the FD and MT genomes while they were more vertical in the other cases.

### Secretory proteins evolve faster than non-secretory proteins

The non-synonymous mutation rate (Ka) of secretory proteins was found to be higher than that of the non-secretory proteins except in one pair (*Frankia* CcI/EuI) where it was equal. The Ka/Ks ratio reflects the rate of adaptive evolution against the background rate. This parameter has been widely studied in the analysis of adaptive molecular evolution, and is regarded as a general method of measuring the rate of sequence evolution. To assess the intensity of mutational constraints, we have considered all of the genes belonging to the core genome for all studied strains of *Frankia, Mycobacterium and Streptomyces*. When these core genes of all *Frankia, Mycobacterium* and *Streptomyces* genomes were studied in all possible pairwise combinations separately for each genus for evolutionary rate analysis, we did find statistically significant differences in Ka/Ks ratios between the secretory and non-secretory protein genes (Mann–Whitney U test significance at P < 0.001 level) in *Frankia* ACN/CcI pair and *Frankia* CcI/FD. Complete list of Signal peptide bearing genes belonging to the core genome of *Frankia* along with their annotation is provided in Additional file 1: Table S1. For the other *Frankia* cases, the differences were not significant. A similar analysis of the *Mycobacterium* genomes showed significant differences with the *M. tuberculosis*/*M. leprae* and in *M. tuberculosis/M. ulcerans* pairings*,* while in *Streptomyces* genomes significant differences with *S. coelicolor*/*S. scabies* and *S. avermitilis/ S. scabies* pairings were found*.* Interestingly, all of the *Frankia* and *Mycobacterium* and some of the *Streptomyces* genomes, which showed significant evolutionary rate differences between secretory and non-secretory protein genes, were either pathogenic, marginally free-living facultative symbiont or at least partly free-living facultative (for grouping refer to Table 1, 2 and 3). This observation prompted us to study these genomes in greater details through pairwise Ka/Ks ratio analysis of all the orthologous genes; both core and non-core (please refer to ‘Secretory protein vs. non-secretory protein in Pairwise comparison’ section).

The normal distribution (Gaussian) curve of the Ka/Ks value for the Secretory protein genes is somewhat skewed (data not shown). The skew of the Ka/Ks in the case of secretory proteins may be associated with biochemical adaptations to the environment. There have been many instances where Ka/Ks values were found to be skewed. For instance, secreted proteins were found to be under low purifying selection in human-mouse sequence alignments [40]. On the other hand, essential genes of *E. coli* were shown to be under strong purifying selection [41] while on the contrary, in the case of plant R genes [42], CHIK envelope proteins [43] and *Shigella* effector gene [44], diversifying selection was shown. In some cases like flu virus HA protein, both purifying and diversifying selection occur at the same time in different sites [45].

### Signal peptides evolve faster than mature regions

A secretory protein is functional only when it reaches the appropriate cellular compartment. The translocation of secretory proteins across the bacterial cytoplasmic membrane can be mediated by N-terminal signal peptides. After translocation across the membrane, signal peptides are normally cleaved from the preprotein by signal peptidases and it has even been suggested signal peptides may end up in the membrane there to play a role unrelated to that of the rest of the proteins [46]. Numerous analyses have indicated that there are considerable rate variations among genes and across different gene regions or subdomains [47]. This suggests that signal peptide (SP) parts might have rates of molecular evolution that are different from that of the mature peptide (MP) parts. In all possible *Frankia* pairs, significant differences were found in the degree of evolutionary change (i.e. Ka/Ks) between SP and MP (Mann–Whitney U test, P < 0.001) (Table 5). However, the *Frankia* ACN/EAN, and ACN/Eul pairings showed more prominent differences between signal peptide and mature parts. Similar trends were also observed among the *Mycobacterium* and *Streptomyces* genomes. In many cases, the Ka/Ks values of signal peptides were found to be 2–7 times higher than those of the mature proteins. Similar results for an increased rate of evolution of signal peptides were reported for yeast [48] and avian growth hormone genes [49]. Although there might be a tendency, we failed to find a strong correlation between Ka/Ks values of the mature and signal peptides. For all of our datasets, the Ka/Ks value of the signal peptide was found to strongly co-vary with Ka/Ks value of the entire peptide. Thus, it seems that the rate of evolution of the entire peptide may be correlated with the rate of evolution of the signal peptide.

**Table 5:** The rate of synonymous (Ks) and non-synonymous (Ka) nucleotide substitution for secretory (signal peptide and mature peptide) and non-secretory proteomes

	**Ka (±S. E.)**	**Ks (±S. E.)**	**Ka/Ks (±S. E.)**
***Frankia *ACN/ CcI**			
Signal Peptide (SP)	0.897 ± 0.335	11.698 ± 2. 627	0.182 ± 0.031
Mature Peptide (MP)	0.151 ± 0.009	10.499 ± 3. 11	0.066 ± 0.005
Secretory Protein (Sec)	0.105 ± 0.006	6.893 ± 2. 006	0.051 ± 0.003
Non-secretory Protein (NSec)	0.083 ± 0.002	6.555 ± 0.614	0.043 ± 0.001
***Frankia *ACN/ FD**			
Signal Peptide (SP)	0.5656 ± 0.2004	24.4256 ± 14.1669	0.1952 ± 0.063
Mature Peptide (MP)	0.2370 ± 0.0328	12.1243 ± 9.3627	0.0980 ± 0.028
Secretory Protein (Sec)	0.2690 ± 0.0255	27.415 ± 10.51547	0.077 ± 0.0211
Non-secretory Protein (NSec)	0.1862 ± 0.0028	31.77567 ± 1.283	0.0336 ± 0.001
***Frankia *ACN/ Ean**			
Signal Peptide (SP)	2.14 ± 0.6975	23.82 ± 4. 32	0.479 ± 0.262
Mature Peptide (MP)	0.194 ± 0.0124	29.85 ± 6. 224	0.0363 ± 0.005
Secretory Protein (Sec)	0.183 ± 0.008	29.785 ± 4. 460	0.040 ± 0.004
Non-secretory Protein (NSec)	0.144 ± 0.002	23.688 ± 1. 120	0.036 ± 0.001
***Frankia *ACN/ Eul**			
Signal Peptide (SP)	2. 99 ± 0.754	29. 198 ± 4. 055	0.568 ± 0.211
Mature Peptide (MP)	0.258 ± 0.011	50.244 ± 6. 94	0.035 ± 0.004
Secretory Protein (Sec)	0.235 ± 0.008	36. 971 ± 4. 836	0.043 ±0.004
Non-secretory Protein (NSec)	0.178 ± 0.003	33. 228 ± 1. 306	0.035 ± 0.001
***Frankia *CcI/FD**			
Signal Peptide (SP)	0.3762 ± 0.0565	5.4064 ± 2.5889	0.2136 ± 0.0524
Mature Peptide (MP)	0.2367 ± 0.0208	19.3491 ± 9.5834	0.0581 ± 0.0104
Secretory Protein (Sec)	0.265 ± 0.02113	18.01 ± 7.5958	0.0633 ± 0.0103
Non-secretory Protein (NSec)	0.1901 ± 0.003	28.1726 ± 1.158	0.0334 ± 0.001
***Frankia *CcI/ Ean**			
Signal Peptide (SP)	0.451 ± 0.125	12.861 ± 6.345	0.139 ± 0.054
Mature Peptide (MP)	0.206 ± 0.015	23.179 ± 10.192	0.043 ± 0.008
Secretory Protein (Sec)	0.179 ± 0.010	35.333 ± 4. 793	0.027 ± 0.003
Non-secretory Protein (NSec)	0.147 ± 0.002	24.320 ± 1. 059	0.031 ± 0.001
***Frankia *CcI/ Eu1**			
Signal Peptide (SP)	2.202 ± 1. 866	13.475 ± 8. 425	0.282 ± 0.111
Mature Peptide (MP)	0.143 ± 0.025	21.280 ± 9. 465	0.070 ± 0.014
Secretory Protein (Sec)	0.097 ± 0.006	5.761 ± 2. 462	0.061 ± 0.005
Non-secretory Protein (NSec)	0.097 ± 0.015	5.244 ± 0.511	0.047 ± 0.001
***Frankia *FD/ Eu1**			
Signal Peptide (SP)	0.450 ± 0.125	12.86 ± 6.34	0.138 ± 0.053
Mature Peptide (MP)	0.205 ± 0.015	23.179 ± 10.19	0.0429 ± 0.008
Secretory Protein (Sec)	0.307 ± 0.015	30.9 ± 10.99	0.0502 ± 0.0125
Non-secretory Protein (NSec)	0.189 ± 0.002	25.7 ± 1.32	0.0288 ± 0.009
***Frankia *FD/ Ean**			
Signal Peptide (SP)	2.202 ± 1.865	13.475 ± 8.4	0.282 ± 0.11
Mature Peptide (MP)	0.143 ± 0.022	21.28 ± 5.96	0.070 ± 0.011
Secretory Protein (Sec)	0.329 ± 0.016	29.094 ±10.111	0.053 ± 0.011
Non-secretory Protein (NSec)	0.203 ± .002	23.128 ± 8.98	0.028 ± .009
***Frankia *Ean/ Eul**			
Signal Peptide (SP)	0.677 ± 0.037	20.845 ± 8. 942	0.321 ± 0.034
Mature Peptide (MP)	0.235 ± 0.021	23.780 ± 7. 688	0.065 ± 0.005
Secretory Protein (Sec)	0.230 ± 0.009	51.172 ± 5. 025	0.033 ± 0.001
Non-secretory Protein (NSec)	0.201 ± 0.013	32.649 ± 1. 250	0.032 ± 0.004
***S. coelicolor/S. avermitilis***			
Signal Peptide (SP)	0.483 ± 0.157	10.529 ± 2. 48	0.166 ±0.016
Mature Peptide (MP)	0.144 ± 0.005	11.029 ± 3. 42	0.061 ± 0.003
Secretory Protein (Sec)	0.142 ± 0.005	13.460 ± 3. 072	0.066 ± 0.003
Non-secretory Protein (NSec)	0.109 ± 0.002	10.344 ± 0.601	0.052 ± 0.001
***S. coelicolor/S. griseus***			
Signal Peptide (SP)	0.952 ± 0.229	26.889 ± 3.260	0.157 ± 0.0250
Mature Peptide (MP)	0.210 ± 0.007	28.96 ± 5.938	0.049 ±0.003
Secretory Protein (Sec)	0.226 ± 0.007	39.273 ± 4.598	0.048 ± 0.004
Non-secretory Protein (NSec)	0.166 ± 0.002	27.224 ± 0.941	0.043 ± 0.001
***S. coelicolor- S. somaliensis***			
Signal Peptide (SP)	1.648 ± 0.439	26.224 ± 5.183	0.324 ± 0.113
Mature Peptide (MP)	0.227 ± 0.009	35.567 ± 3.675	0.033 ± 0.005
Secretory Protein (Sec)	0.235 ± 0.005	45.582 ± 7.275	0.041 ± 0.005
Non-secretory Protein (NSec)	0.151 ± 0.002	26.060 ± 1.209	0.040 ± 0.001
***S. coelicolor- S. scabiei***			
Signal Peptide (SP)	0.585 ± 0.126	15.974 ± 2.126	0.177 ± 0.016
Mature Peptide (MP)	0.172 ± 0.005	18.214 ± 3.100	0.057 ± 0.003
Secretory Protein (Sec)	0.159 ± 0.008	15.480 ± 4.913	0.067 ± 0.005
Non-secretory Protein (NSec)	0.103 ± 0.002	10.699 ± 0.802	0.050 ± 0.001
***S. avermitilis- S. somaliensis***			
Signal Peptide (SP)	0.634 ± 0.101	23.098 ± 4.180	0.347 ± 0.088
Mature Peptide (MP)	0.233 ± 0.009	43.112 ± 6.251	0.040 ± 0.005
Secretory Protein (Sec)	0.236 ± 0.008	41.932 ± 6.196	0.042 ± 0.005
Non-secretory Protein (NSec)	0.147 ± 0.002	27.533 ± 1.286	0.036 ± 0.001
***S. avermitilis- S. scabiei***			
Signal Peptide (SP)	0.554 ± 0.146	12.172 ± 1.912	0.268 ± 0.038
Mature Peptide (MP)	0.164 ± 0.005	13.605 ± 2.619	0.068 ± 0.003
Secretory Protein (Sec)	0.134 ± 0.006	3.663 ± 1.879	0.084 ± 0.005
Non-secretory Protein (NSec)	0.096 ± 0.002	7.412 ± 0.719	0.055 ± 0.001
***S. avermitilis/ S. griseus***			
Signal Peptide (SP)	1.359 ± 0.326	21.298 ± 2.755	0.504 ± 0.036
Mature Peptide (MP)	0.210 ± 0.007	48.403 ± 6.312	0.037 ± 0.003
Secretory Protein (Sec)	0.234 ± 0.006	44. 581 ± 4.500	0.047 ± 0.005
Non-secretory Protein (NSec)	0.158 ± 0.002	26. 284 ± 0.889	0.040 ± 0.001
***S. somaliensis- S. scabiei***			
Signal Peptide (SP)	1.346 ± 0.450	25.264 ± 4.391	0.492 ± 0.283
Mature Peptide (MP)	0.223 ± 0.007	24.696 ± 5.321	0.032 ± 0.004
Secretory Protein (Sec)	0.232 ± 0.008	42.124 ± 6.385	0.056 ± 0.006
Non-secretory Protein (NSec)	0.151 ± 0.002	27.537 ± 1.294	0.035 ± 0.004
***S. somaliensis- S. griseus***			
Signal Peptide (SP)	0.565 ± 0.145	20.919 ± 3.434	0.170 ± 0.026
Mature Peptide (MP)	0.220 ± 0.009	42.007 ± 6.413	0.034 ± 0.004
Secretory Protein (Sec)	0.197 ± 0.006	38.510 ± 6.153	0.043 ± 0.005
Non-secretory Protein (NSec)	0.157 ± 0.039	20.710 ± 3.643	0.041 ± 0.003
***S. scabiei- S. griseus***			
Signal Peptide (SP)	0.734 ± 0.178	22.970 ± 2.788	0.743 ± 0.256
Mature Peptide (MP)	0.216 ± 0.007	49.319 ± 6.226	0.037 ± 0.003
Secretory Protein (Sec)	0.211 ± 0.008	36.526 ± 6.828	0.046 ± 0.005
Non-secretory Protein (NSec)	0.148 ± 0.002	24.148 ± 1.233	0.040 ± 0.001
***M. tuberculosis/M. leprae***			
Signal Peptide (SP)	0.414 ± 0.0408	12.978 ± 6.284	0.165 ± 0.069
Mature Peptide (MP)	0.111 ± 0.011	1.406 ± 0.055	0.079 ± 0.008
Secretory Protein (Sec)	0.137 ± 0.009	1.4109 ± 0.076	0.099 ± 0.008
Non-secretory Protein (NSec)	0.122 ± 0.002	2.173 ± 0.288	0.088 ± 0.001
***M. tuberculosis/M. smegmatis***			
Signal Peptide (SP)	1.059 ± 0.273	30.888 ± 8.194	0.098 ± 0.031
Mature Peptide (MP)	0.199 ± 0.021	23.071 ± 10.099	0.043 ± 0.014
Secretory Protein (Sec)	0.239 ± 0.017	39.868 ± 9.856	0.032 ± 0.0105
Non-secretory Protein (NSec)	0.163 ± 0.003	28.329 ± 1.413	0.030 ± 0.001
***M. tuberculosis/ M. ulcerans***			
Signal Peptide (SP)	0.685 ± 0.258	8.146 ± 3.966	0.436 ± 0.159
Mature Peptide (MP)	0.109 ± 0.014	5.997 ± 4.625	0.069 ± 0.010
Secretory Protein (Sec)	0.127 ± 0.012	1.48434 ± 0.077	0.0864 ± 0.007
Non-secretory Protein (NSec)	0.096 ± 0.002	2.616 ± 0.353	0.065 ± 0.001
***M. tuberculosis/ M. vanbaalenii***			
Signal Peptide (SP)	0.821 ± 0.083	13.294 ± 5.633	0.247 ± 0.072
Mature Peptide (MP)	0.226 ± 0.018	21.208 ± 9.415	0.053 ± 0.012
Secretory Protein (Sec)	0.247 ± 0.015	20.711 ± 7.419	0.049 ± 0.009
Non-secretory Protein (NSec)	0.168 ± 0.003	26.060 ± 1.344	0.032 ± 0.001
***M. leprae/ M. smegmatis***			
Signal Peptide (SP)	1.003 ± 0.129	20.464 ± 6.323	0.207 ± 0.062
Mature Peptide (MP)	0.247 ± 0.025	16.558 ± 5.325	0.0278 ± 0.005
Secretory Protein (Sec)	0.281 ± 0.018	34.78545 ± 6.822	0.02763 ± 0.006
Non-secretory Protein (NSec)	0.188 ± 0.003	28.210 ± 1.291	0.026 ± 0.001
***M. leprae/ M. ulcerans***			
Signal Peptide (SP)	0.462 ± 0.056	14.975 ± 6.791	0.273 ± 0.080
Mature Peptide (MP)	0.158 ± 0.021	6.794 ± 4.753	0.0731 ± 0.009
Secretory Protein (Sec)	0.176 ± 0.020	5.359 ± 3.338	0.081 ± 0.007
Non-secretory Protein (NSec)	0.132 ± 0.003	2.877 ± 0.312	0.070 ± 0.002
***M. leprae/ M. vanbalanii***			
Signal Peptide (SP)	1.322 ± 0.349	33.707 ± 8.948	0.115 ± 0.048
Mature Peptide (MP)	0.278 ± 0.0267	17.136 ± 6.912	0.038 ± 0.006
Secretory Protein (Sec)	0.302 ± 0.021	25.442 ± 6.775	0.041 ± 0.008
Non-secretory Protein (NSec)	0.193 ± 0.004	25.629 ± 1.238	0.029 ± 0.001
***M. smegmatis / M. ulcerans***			
Signal Peptide (SP)	1.409 ± 0.415	18.212 ± 5.189	0.293 ± 0.138
Mature Peptide (MP)	0.225 ± 0.0184	18.889 ± 7.0584	0.041947 ± 0.007
Secretory Protein (Sec)	0.257 ± 0.017	27.432 ± 6.438	0.037 ± 0.006
Non-secretory Protein (NSec)	0.163 ± 0.003	28.286 ± 1.443	0.030008 ± 0.001
***M. smegmatis / M. vanbaalenii***			
Signal Peptide (SP)	3.458 ± 1.548	19.415 ± 5.519	0.668 ± 0.356
Mature Peptide (MP)	0.1737 ± 0.014936	19.717 ± 8.35107	0.0518 ± 0.0084
Secretory Protein (Sec)	0.2046 ± 0.01668	23.418 ± 7.994028	0.0621 ± 0.0111
Non-secretory Protein (NSec)	0.1252 ± 0.0027	16.09933 ± 1.215	0.0416 ± 0.0014
***M. ulcerans / M. vanbaalenii***			
Signal Peptide (SP)	1.640 ± 0.833	20.487 ± 5.451	0.1985 ± 0.0625
Mature Peptide (MP)	0.2575 ± 0.017255	13.6171 ± 4.540	0.0524 ± 0.0084
Secretory Protein (Sec)	0.2721 ± 0.0158	24.692 ± 6.523	0.0438 ± 0.0071
Non-secretory Protein (NSec)	0.165 ± 0.0031	27.909 ± 1.427	0.0309 ± 0.0011

### Distribution into COGs

In order to detect if the core genome and conserved secretome had similar contents, they were distributed into functional categories (COGs) and compared with the whole genome of two representative strains for the three genera *Frankia, Streptomyces* and *Mycobacterium*. It thus seems that the two intracellular bacteria (MT and FD) shared a similar distribution of their secretomes into COGs. The full genomes have similar tendencies in that the pairs of genomes belonging to the three genera were close to one another especially *Streptomyces* and *Frankia* and associated with categories I (lipid transport) in the case of *Mycobacterium*, with categories P (Inorganic ion transport and metabolism) and C (Energy production and conversion) in the case of *Frankia* and with categories T (signaling), K (transcription) and V (defense) in the case of *Streptomyces*. When core secretomes were considered, the three pairs were not maintained with the three strains (MT, FD and SS) comprising pathogens being close to one another while the saprophytes were more distant (Figure 1). With regards to their secreted proteomes, *Frankia* FD and *Mycobacterium* MT were closer to one another than either was to *Streptomyces* (Figure 1).

### Codon usage bias affecting the selection pressure

**Figure 2:**
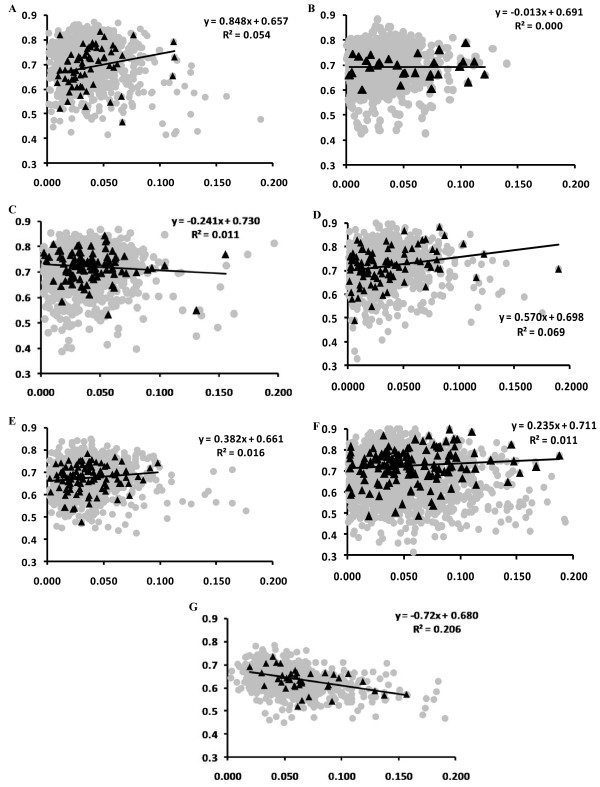
**Scatter plot of Ka/Ks value versus Codon Adaptation index (CAI) in various Actinobacteria (left to right &Top to bottom) (A) *Frankia *EAN1pec (B) *Frankia *FD (C) *Frankia *ACN14a (D) *Frankia *Eul1c (E) *Frankia *CcI3 (F) *S. coelicolor *(G) *M. tuberculosis*.** The x-axis represents the Ka/Ks value and y-axis represents CAI value.

We examined whether evolutionary constraints on the genes are influenced by the codon usage bias. For *Frankia* ACN and *Frankia* FD (Figure 2), the evolutionary rate, particularly the Ka/Ks ratio, was negatively correlated to CAI values for all of the genes belonging to the core including the secretory protein genes (Pearson correlation coefficient, R = -0.017 for core genes and R = -0.16 for secretory protein genes). Similar trends were also found with the *Mycobacterium* strains. One explanation for this negative correlation is that codon usage bias correlated positively with the intensity of purifying selection [50]. Therefore, genes with a stronger codon usage bias (i.e. with high CAI value) will undergo higher negative selection pressure and thus, the evolutionary rate will be slower at non-synonymous or synonymous sites. On the other hand, *Frankia* strains CcI, EAN, and Eul, and *Streptomyces*, showed a negative correlation between Ka/Ks ratio and CAI values for the core genes as a whole, while the secretory proteins exhibited a reverse trend (*i.e.* the Ka/Ks ratio was positively correlated to CAI, with R values ranging from 0.188 to 0.262). This kind of unusual relationship between evolutionary rate and CAI value in signal-peptide-bearing genes was reported earlier for *Streptomyces*[48]. They have proposed that intensity of purifying selection was significantly relaxed in such genes.

### Secretory protein vs. non-secretory protein in pairwise comparison

Various combinations of *Frankia, Mycobacterium and Streptomyces* genes were used for pairwise calculation of Ka/Ks. For this analysis, we have first screened out the orthologous gene pairs between genome pairs and then calculated the Ka/Ks value for all orthologous gene pairs. From these data, the secretory protein genes were identified as those predicted to have a signal peptide in both members of the orthologous pair. Their Ka/Ks values were compared to the rest of the genes. Average Ka/Ks values for pair-wise genome comparisons among *Frankia, Mycobacterium* and *Streptomyces* are provided in Additional file 2: Table S2. In Table 6, a matrix format is provided with each cell representing the difference between average Ka/Ks value of secretory protein genes and non-secretory protein genes. Generally among the marginally free-living facultative symbiont (Group C strains) *Frankia* strains (*i.e. Frankia* CcI and FD), the difference in evolutionary rates of secretory proteins and non-secretory proteins was quite robust. The Mann–Whitney U-test showed that the difference was highly significant (P < 0.001 in a two-tailed test). Similarly, in all combinations of *Frankia* ACN, CcI and FD also showed statistically significant differences. Whereas, in other pairing with *Frankia* ACN and the two *Elaeagnus*-infecting strains, which are predominantly free-living facultative symbionts (Group B strains), showed no significant differences in the Ka/Ks values of secretory proteins and non-secretory proteins with the exception of the EAN–CcI pairwise combination. Here, the difference in Ka/Ks ratios of secretary and non-secretary proteins was significant at p < 0.05. A trend was also observed for the other actinobacteria strains analyzed. Only pairing comprised of pathogenic *Mycobacterium* strains (*i.e. M. tuberculosis, M. leprae* and *M. ulcerans*) showed a significant difference between the evolutionary rate of secretory and non-secretory proteins. The Ka/Ks ratio of secretory and non-secretory proteins was not significant for pairing among non-pathogenic strains (like *M. vanbaalenii* and *M. smegmati*s) or with a combination of pathogenic and non-pathogenic strain (like *M. tuberculosis/M. vanbaalenii*, or *M. leprae/M. smegmatis*)*.* Analysis of the *Streptomyces* pairing also showed significant differences in the Ka/Ks ratio of secretory and non-secretory proteins in pairs like *S. coelicolor*/*S. scabies, S. avermitilis/ S. scabies and S. scabies/S. somaliensis*. Incidentally, *S. scabies and S. somaliensis* are the pathogenic strains of *Streptomyces.*

**Table 6:** Pairwise comparison of Ka/Ks ratio in various strains of studied genera

**Strains**	**Ka/Ks ratio between various pairs**				
*Frankia*	F1 (Fd)♠	F2 (Fc)♠	F3 (Fa)♣	F4 (Fu)♥	F5 (Fe)♥
F1 (datisca)♠	-	0.059**	0.038**	0.015	0.003
F2 (CcI3)♠		-	0.026**	0.005	0.027*
F3 (ACN14a)♣			-	0.013	0.020
F4 (EuI1b)♥				-	0.008
F5 (EaN1pec)♥					-
*Mycobacterium*	M1 (Mt)♦	M2 (Ml)♦	M3 (Mu)♦	M4 (Ms)♪	M5 (Mv)♪
M1 (*tuberculosis*)♦	-	0.034**	0.023**	0.000	0.002
M2 (*leprae*)♦		-	0.013**	0.007	0.002
M3 (*ulcerans*)♦			-	0.004	0.003
M4 (*smegmatis*) ♪				-	0.01
M5 (*vanbaalenii*) ♪					-
*Streptomyces*	S1 (Ss)♦	S2 (So)♦	S3 (Sc)♪	S4 (Sa)♪	S5 (Sg)♪
S1 (*scabiei*)♦	-	0.016**	0.018**	0.018**	0.003
S2 (*somaliensis*)♦		-	0.008	0.011	0.002
S3 (*coelicolor*)♪			-	0.012	0.005
S4 (*avermitilis*)♪				-	0.007
S5 (*griseus*)♪					-

Strains of *Frankia*: Symbiont of *Datisca glomerata* (F1), CcI3 (F2), ACN14a (F3), Eul1c (F4), EAN1pec (F5); Strains of *Mycobacterium*: *M. tuberculosis* CDC1551 (M1), *M. leprae* TN (M2), *M. ulcerans* Agy99 (M3), *M. smegmatis* MC2 155 (M4), *M. vanbaalenii* PYR-1(M5); Strains of *Streptomyces* : (2) *S. scabiei* (S1), *S. somaliensis* (S2), *S. coelicolor* A3(S3), *S. avermitilis* MA-4680 (S4) and *S. griseus griseus* NBRC 13350 (S5) Each cell represents the difference between Average Ka/Ks value of secretory protein genes and non-secretory protein genes. *Symbols*: *=Significant @ p<0.05 level; **=Significant @ p<0.01 level; ♥=Predominantly free-living Facultative symbiont (GroupA); ♣=Partly free-living Facultative symbiont (GroupB); ♠=Marginally free-living / Obligate symbiont (GroupC); ♦=Pathogen; ♪=Non pathogen.

These above results in total indicate an overall trend that the evolutionary constraints on secretory proteins as a whole in marginally free-living facultative symbiont or pathogenic strains were significantly increased compared to those occurring in saprophytic or free-living organisms. A possible explanation for this trend is that high Ka/Ks ratios of secretory proteins in pathogens and symbionts may reflect adaptive evolution of their sequences.

## Conclusions

A definite trend emerged from our analysis of the evolutionary rates and patterns for various gene types among five *Frankia*, five *Mycobacterium* and five *Streptomyces* genomes. Secretory protein genes for obligate symbionts, marginally free-living facultative symbionts or pathogenic organisms, evolved significantly faster than non-secretory protein genes, whereas genomes of saprophytes or predominantly free-living facultative symbionts did exhibit significant changes in rate. This difference may be a telling genomic signature of loss of autonomy. Although robust purifying selection was encountered in most of the analyses, the secretory protein genes were found to be under stronger evolutionary selection pressure than non-secretary protein genes in symbiotic and pathogenic strains. This difference could be an adaptive strategy for them to interact better with their hosts. Further, within the secretory protein genes, the evolution rate (*K*a/*K*s) of signal peptide, on average, was 2–7 times higher than that of mature proteins. This result suggests that signal peptides might be under relaxed purifying selection. Codon usage analysis of actinobacterial strains under host selection pressure (such as symbiotic *Frankia*, ACN, FD and the pathogenic *Mycobacterium*) suggests that codon usage bias had a negative impact on the selective pressure exerted on the secretory protein genes. These organisms remain in continuous cross-talk with their host particularly through the signal peptides. It thus appears symbiotic and pathogenic bacteria try to remain in a discrete expression mode to avoid elicitation of host defense responses, while concurrently accumulating evolutionary neutral synonymous substitutions.

The expected arrival of a large number of genomes, in particular in genus *Frankia* and relatives, may yield more closely related genomes on which to calculate a larger number of conserved genes than is possible in strains with different host infectivity spectra that have diverged for several millions of years with a reduced core genome. This should help identify proteins and domains subject to strong evolutionary constraints, in particular in lineages where little or no isolates are available among which those determinants involved in symbiotic interactions.

## Methods

### Selection of genomes used in this study

The nucleotide sequences along with their deduced amino acid sequences for all the protein coding gene sequences of five *Frankia* strains namely ACN14a (NC_008278), CcI3 (NC_007777), EAN1pec(NC_009921), EuI1c(NC_014666) and symbiont of *Datisca glomerata* (CP002801) and hereafter will be referred to as ACN, CcI, EAN, EuI and FD respectively along with five *Streptomyces* strains : *S. coelicolor* A3(2)(NC_003888)*, S. avermitilis* MA-4680(NC_003155), *S. griseus* NBRC 13350 (NC_010572), *S. scabiei* NC_013929.1, *S. somaliensis* AJJM01000000 [12] and five *Mycobacterium* strains : *M. leprae* TN (NC_002677), *M. tuberculosis* CDC1551(NC_002755), *M. ulcerans* Agy99 (NC_008611.1), *M. smegmatis* MC2 155 (NC_008596) and *M. vanbaaleni* PYR-1(NC_008726); were downloaded from the JGI-IMG Database (http://img.jgi.doe.gov/cgi-bin/w/main.cgi).

### Identification of orthologous genes

Orthologous genes were identified based on the Reciprocal Best Hits (RBH) approach on amino acid sequences for all the protein coding gene sequences with an E-value threshold of 1e^-10^; an identity ≥ 50% over at least 50% of the alignable region. This approach and parameters had been used previously for screening orthologs in *Streptomyces*[51].

### Identification of secretory protein genes

Secretory protein genes belonging to the core genome were identified using the SignalP 3.0 [52] and TMHMM 2.0 [53] software. Only those genes predicted as secretory proteins by both artificial neural networks and hidden Markov models were selected. Sequences predicted to contain a signal peptide by SignalP were analyzed with TMHMM 2.0 to determine the number of transmembrane (TM) domains. Those having 0–2 transmembrane domains were further considered as done by Mastronunzio et al. [12]. Individual examination of selected genes was made to ensure only genes with viable peptide leader were selected. For the comparison of evolutionary rates of the mature part and the signal peptide part, a dataset of orthologs which signal peptide cleavage site have been detected in both entities was compiled. Mature peptides (complete sequence minus signal peptide) were analyzed by editing out the predicted signal peptide from the alignment file using a Perl script developed by us.

### Evolutionary rate analysis

Orthologous gene alignments were utilized for evolutionary rate analyses. The number of nonsynonymous or synonymous substitutions per site (Ka or Ks, respectively) and their ratio (Ka/Ks) was estimated with Codeml in the PAML software program [54]. A bioperl script was used with the PAML program to estimate the pairwise Ka and Ks values. The script first translated cDNAs into proteins and aligned the protein sequences. The protein alignments were projected back into cDNA coordinates and used by the PAML module to calculate the Ka/Ks ratio using the maximum likelihood method. To study the evolutionary rate of the signal peptide part and the mature part of a protein, the Ka/Ks value of each component of the protein was determined separately.

### Codon bias analysis

Codon adaptation index (CAI) is a measure of directional synonymous codon usage bias [55]. The index uses a reference set of highly expressed genes from a species to assess the relative usage of each codon, and the score of each gene is calculated from the frequency of use of all codons in that gene. The index assesses the extent to which selection has been effective in molding the pattern of codon usage. The CAI value for each gene belonging to core genome was calculated with the help of CAI Calculator 2 (http://userpages.umbc.edu/~wug1/codon/cai/cais.php) [56].

## Competing interest

The authors declare they have no financial or non-financial competing interest of any sort.

## Authors’ contributions

VD and ST performed preliminary analyses, PN, LST and AS conceived the experimental design, ST and AS performed data analyses, PN and AS drafted the manuscript. All authors prepared the final manuscript and approved the final version.

## Supplementary Material

Additional file 1: Table S1 Signal peptide bearing genes belonging to the core genome of *Frankia, Mycobacterium* and *Streptomyces*. The COG category to which these belong was obtained from the IMGer site (https://img.jgi.doe.gov/cgi-bin/er/main.cgi). Click here for file

Additional file 2: Table S2 Average Ka/Ks values for pair-wise genomes comparisons between *Frankia, Mycobacterium* and *Streptomyces*. The Ka/Ks was computed for secreted proteins (presence of a peptide leader) and for other proteins, and the difference between the two was analyzed for significance by the Mann–Whitney test. Click here for file
